# Multi-criteria decision analysis of breast cancer control in low- and middle- income countries: development of a rating tool for policy makers

**DOI:** 10.1186/1478-7547-12-13

**Published:** 2014-05-17

**Authors:** Kristie Venhorst, Sten G Zelle, Noor Tromp, Jeremy A Lauer

**Affiliations:** 1 Department of Primary and Community Care, Radboud University Medical Center, Nijmegen, The Netherlands; 2 Knowledge Institute of Medical Specialists, Utrecht, The Netherlands; 3 Costs, Effectiveness, Expenditure and Priority Setting, World Health Organization, Geneva, Switzerland

**Keywords:** Multi-criteria decision analysis, Priority setting, Breast cancer

## Abstract

**Background:**

The objective of this study was to develop a rating tool for policy makers to prioritize breast cancer interventions in low- and middle- income countries (LMICs), based on a simple multi-criteria decision analysis (MCDA) approach. The definition and identification of criteria play a key role in MCDA, and our rating tool could be used as part of a broader priority setting exercise in a local setting. This tool may contribute to a more transparent priority-setting process and fairer decision-making in future breast cancer policy development.

**Methods:**

First, an expert panel (n = 5) discussed key considerations for tool development. A literature review followed to inventory all relevant criteria and construct an initial set of criteria. A Delphi study was then performed and questionnaires used to discuss a final list of criteria with clear definitions and potential scoring scales. For this Delphi study, multiple breast cancer policy and priority-setting experts from different LMICs were selected and invited by the World Health Organization. Fifteen international experts participated in all three Delphi rounds to assess and evaluate each criterion.

**Results:**

This study resulted in a preliminary rating tool for assessing breast cancer interventions in LMICs. The tool consists of 10 carefully crafted criteria (effectiveness, quality of the evidence, magnitude of individual health impact, acceptability, cost-effectiveness, technical complexity, affordability, safety, geographical coverage, and accessibility), with clear definitions and potential scoring scales.

**Conclusions:**

This study describes the development of a rating tool to assess breast cancer interventions in LMICs. Our tool can offer supporting knowledge for the use or development of rating tools as part of a broader (MCDA based) priority setting exercise in local settings. Further steps for improving the tool are proposed and should lead to its useful adoption in LMICs.

## Background

As the second most common cancer in the world and the most common cancer in women, breast cancer is an important health problem globally [1]. Although it was originally considered to be a disease of the developed world, low- and middle-income countries (LMICs) are experiencing large increases in incidence [2]. Mortality-to-incidence rates remain relatively high in these areas [3], possibly due to relatively poor breast cancer control strategies (e.g. awareness raising, early detection, treatment) and differences in cultural beliefs [2,4]. Because strong early detection programs are beneficial, the World Health Organization (WHO) seeks to improve appropriate breast cancer control programs in LMICs.

Cost-effectiveness analyses (CEAs), based on the maximization of health benefits, have often been used for the selection of breast cancer control strategies. To provide an evidence base for the cost-effectiveness of breast cancer interventions in LMICs, a consortium of the WHO, Erasmus University Rotterdam, the Radboud University Nijmegen Medical Center, and the Susan G. Komen for the Cure Foundation initiated an international study in 2010 [5]. Such CEAs may help governments decide how to spend scarce health care resources more efficiently. However, decision-makers often deviate from CEA results because other principles such as equal treatment and priority to the worst-off [6-8] and other factors like feasibility or acceptability influence decisions, as well [9-11]. Ignorance about these criteria may induce implementation problems or inequality among certain patient groups [12-14].

Multi Criteria Decision Analysis (MCDA) is a well-accepted framework that can simultaneously assess multiple criteria for priority setting of interventions [15]. Different approaches of MCDA are proposed but contain at least the following elements: 1) selection of relevant interventions, 2) selection of criteria for priority setting, 3) collecting evidence and rating the performance of interventions on selected criteria, 4) deliberation on the evidence and performance of interventions with the aim to select the best interventions for implementation [16-19].

Several studies have shown the potential of MCDA in prioritizing health interventions, however, it has not yet been applied for the selection of breast cancer control interventions [20-23]. Recently, MCDA has been criticized for being technocratic and conceptually challenging for local decision makers [24]. Therefore, the development of a tool to support local policy makers in selecting criteria and in rating the performance of interventions on these criteria is warranted.

The objective of this study is to develop a rating tool to assess breast cancer interventions along the continuum of care, within the context of the overarching breast cancer CEA project [5]. The rating tool will be composed of criteria, criteria definitions, criteria weights and rating scales to measure the overall impact of breast cancer interventions and support the priority setting process. Such a rating tool can be used in a broader, MCDA based, priority setting process to develop cancer control strategies in a local setting.

## Methods

To develop the rating tool we established an expert panel (n = 5) of breast cancer and priority-setting experts from WHO and the Radboud University Nijmegen Medical Centre. The expert panel consisted of two health economists, a scientific researcher, a public health specialist and a student on health technology assessment. Three of the experts are co-authors of this article (KV, SZ and JL). A detailed overview of the considerations made by the expert panel in the development of the rating tool is provided as additional information (Additional file 1). Below we describe the most important steps that were taken to develop the tool.

A literature search using PubMed and Google Scholar was performed for the identification of a first set of predefined criteria. Different combinations of the terms ‘criteria’, ‘values’, ‘factors’, ‘priority setting’, ‘decision making’, and ‘policy making’ were used as the query. The expert panel discussed the list in order to avoid overlap among the criteria. For the remaining criteria, clear definitions were defined with the help of glossaries and documents published by the WHO [25-27].

To develop the scoring scales, another literature study was performed for each criterion of this predefined list. When no or little information was available, scoring scales were mainly based on discussions with the expert panel.

### The Delphi study

The list of predefined criteria and scoring scales was further refined by the opinion of experts from all over the world. A Delphi study was chosen because of the anonymity of participants, the opportunity to include participants globally, and the time and money available to conduct the study [28]. Delphi studies have proven to be appropriate for finding a core list of evaluation criteria [29].

#### Participants

Experts were selected following WHO selection criteria that include a balanced geographical and gender representation, expertise in the technical area (particularly in LMICs), and absence of any relevant interest in the personal declaration of interest form. Twenty-nine experts with expertise in priority setting or breast cancer policies in LMICs were involved, ensuring methodological as well as substantive quality. Experts were identified by approaching authors of relevant articles and by snowball sampling. Among the experts there were epidemiologists, cancer survivors, pathologists, guideline-developers, public health specialists, radiotherapists, surgeons, researchers, managers, strategists and ethicists.

#### First round

In this round, the list with criteria based on the performed literature study was presented to the participants. The participants were asked to score the criteria on five-point Likert scales, according to whether they agreed that interventions scoring high on the criterion should be more prioritized (1 = strongly disagree, 2 = disagree, 3 = neutral, 4 = agree, 5 = strongly agree). The experts could give comments on the list and mention whether important criteria were missing. In addition, the definitions and scoring scales of the criteria were presented and participants were asked to provide comments. Likert scales were chosen for this first round because this method is reviewed as acceptable for a Delphi study and is simple and easy to perform [30].

#### Second round

This second round showed the scores and comments given in the first round, together with the adaptations to which they had led. Subsequently, participants were asked whether they agreed on the adaptations and if they could clarify their answers.

#### Third round

Based on the proportion of participants agreeing on the adaptations made after the first round and on the comments provided, some final changes were made to the criteria list. These final criteria and their definitions and scoring scales were shown to the participants, who were asked whether they agreed that this final list contained the most relevant criteria for the prioritization of breast cancer interventions. Furthermore, participants were asked to divide 100 points over the criteria according to their relative importance for the evaluation of breast cancer interventions.

#### The analysis

The analysis of the answers was both quantitative and qualitative. After the first Delphi round, mean and median scores on the Likert scales for “the importance” of criteria were calculated. The second round resulted in a percentage of participants who agreed with the suggested adaptations. After the third round, the mean and median weights given according to the importance of criteria were calculated. All participant comments were quantitatively analyzed.

## Results

**Figure 1:**
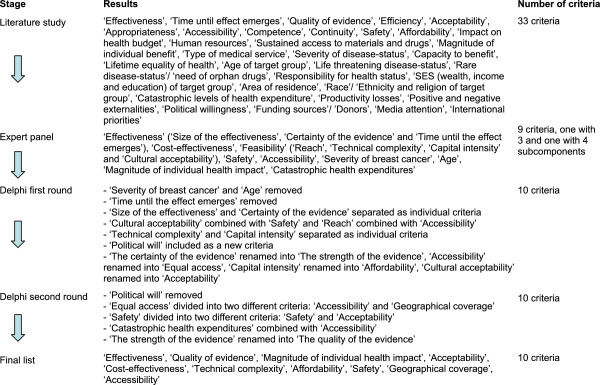
Overview of the development of the criteria list.

The literature search on criteria resulted in a total of 33 criteria (Figure 1). After the expert panel discussed these criteria, nine criteria remained for the Delphi study. Two criteria, effectiveness and feasibility, were divided into three and four subcomponents. For each of these nine criteria and the subcomponents, a definition and a potential scoring scale were defined.

### Participants

Out of 72 experts who were asked, 29 were willing to participate. Of these, 17 were experts on priority setting, and 12 were experts on breast cancer policies. The first questionnaire was completed by 23 participants, the second questionnaire by 19 participants, and the third questionnaire by 15 participants. Reasons for not completing a questionnaire were private circumstances and disagreement with the aim of this research (n = 1). Most participants, however, gave no reason.

### First round

Based on the results of the first round, two criteria (‘Severity of breast cancer’ and ‘Age’) and one of the components (‘The time until the effect emerges’) were suggested for removal; two of the components were suggested to be combined with two criteria; and all the other components were suggested for separation into different criteria. Also, a new criterion was suggested (‘Political will’), two definitions were refined, and four scoring scales were adapted. These adaptations led to a list of 10 criteria. For all criteria, except for the criterion ‘Effectiveness’, there was divergence in Likert scale scores. The average and median Likert scale values and most important comments on the criteria are shown in Table 1.

**Table 1:** Initial criteria including Likert scores and important comments given in the Delphi study

	***Average likert scores***	***Median likert scores***	***Range of likert scores***	***Most important comments***
Effectiveness	4.75	5	4-5	Effectiveness is covered by its components. Effectiveness should therefore be removed and its components should be independent criteria, otherwise they will overlap.
Size of effectiveness	4.70	5	3-5	No important comments.
Certainty of the evidence	4.35	5	1-5	Not related to effectiveness only. The strength of the evidence varies by criterion for any given intervention. Much simpler and effective to include considerations of certainty of evidence in assigning scores for all given criterion.
Time until the effect emerges	3.09	3	1-5	Time preference for immediate effects goes against principles of intergenerational equity, and is especially inappropriate for preventive services. Therefore this criterion should be removed.
Cost-effectiveness	4.25	4.5	1-5	MCDA might replace C/E. We can have costs but “effectiveness” is defined by the sum of the criteria so adding this criterion introduces double-counting.
				Efficiency cannot be replaced by costs since higher costs do not per se mean lower efficiency as the effectiveness may be higher.
Feasibility	4.23	4	2-5	This should be four different criteria, otherwise they will overlap each other.
Reach	4.46	5	2-5	See comments accessibility.
Technical complexity	3.5	3.5	1-5	No important comments.
Capital intensity	3.75	4	1-5	This criterion should not be limited to capital costs but also explicitly include operating costs required from the health system.
Cultural acceptability	4.13	4.5	1-5	No important comments.
Safety	4	4	2-5	The importance of safety may vary with respect to whose safety (provider vs. patient) and what is at stake, while the level of acceptability may remain the same. Therefore acceptability and safety should be kept separated.
Accessibility	4.33	4.5	1-5	Accessibility due to geographical coverage of an intervention (‘Reach’) is not the same as accessibility due to socio-economic status. Therefore this criterion should be about equal access for patients with different socio-economic status, while geographical coverage should be covered by another criterion (‘Reach’)
Severity of breast cancer	3.26	3	1-5	Of course I think that palliative care is very important. On the other hand, if you do nothing for all the people with earlier stage cancer, the cancer will progress and they will all need palliative care. So you could treat people with stage 1 or 2 cancer and most of them will not experience late stage cancer, therefore will not need palliative care. I guess I don’t find this a useful way to think about breast cancer.
Age	3.29	3.5	1-5	Ages of patients with breast cancer don’t seem appropriate even if one wanted to create prioritized age groups, which I wouldn’t.
Magnitude of individual health impact	3.83	4	1-5	No important comments.
Catastrophic health expenditures	4.17	5	1-5	Affordability is about whether the health system can afford an intervention and catastrophic health expenditures is about whether patients can afford it. Extreme health expenditures might however be covered by accessibility, because patients with lower socio-economic status cannot afford high health expenditures.

### Second round

Based on the results of the second round, the new suggested criterion (‘Political will’) was removed again because participants argued that political will would also depend on the results of interventions on the other criteria; political will changes too often; and MCDA aims at a more fair priority-setting process while political will might even be clearly unfair. Two criteria (‘Equal access’ and ‘Acceptability’) were separated into two different criteria (‘Geographical coverage’ and ‘Accessibility’; ‘Acceptability’ and ‘Safety’); two criteria were combined (‘Catastrophic health expenditures’ and ‘Acceptability’); and some small refinements to most of the definitions and scoring scales were made. An overview of the changes made in the criteria list is shown in Figure 1. The second round resulted in a final list of 10 criteria (Table 2).

**Table 2:** Final criteria list for the prioritization of breast cancer interventions including weights

	**Definition**	**Potential scoring scales**	Average weight* *(min-max)*	**Median weight**
**Effectiveness**[31-35]	Effectiveness is the extent to which an intervention impacts the most relevant health-related outcomes (e.g. time to recurrence or healthy life years gained). In comparison of effectiveness of interventions, it is important to note that the most relevant health-related outcome should be consistent for all interventions under consideration [25].	Size of the effect (e.g. in a population of 1 million people):	17.33 *(5–50)*	15
		**0** less effective (e.g. < 50 healthy life years gained a year)		
		**1** effective (e.g. ≥ 50 < 100 healthy life years gained a year)		
		**2** very effective (e.g. ≥ 100 healthy life years gained a year) [36]		
**Quality of the evidence**[31,32,35,37,38]	The risk of bias and the extent of the confidence that the evidence is adequate to support a particular decision or recommendation [39].	**0** very little or limited confidence in the evidence: the estimated values may be substantially different from the outcomes in reality	11.93 *(0–20)*	12
		**1** moderately confident about the evidence: The estimated values are likely to be close to the outcomes in reality, but there is a possibility that it is different		
		**2** very confident that the estimated values lie close to the outcomes in reality [39]		
**Magnitude of individual health impact**[32,38]	Interventions offering small benefits for many may be viewed differently from those offering large benefits for a few. When one of the two is preferred above the other, interventions providing the preferred effect (concentrated or dispersed) might be more prioritized [32].	Scoring scale a could be used in the case that local stakeholders decide that large individual health benefits are preferred above helping more people.	8.60 *(0–25)*	10
		Scoring scale b could be used in the case that local stakeholders decide that helping more people is preferred above large individual health benefits.		
		a		
		**0** small individual health impact		
		**1** moderate individual health impact		
		**2** large individual health impact		
		b		
		**0** benefits for just a few people.		
		**1** benefits for a moderate number of people		
		**2** benefits for many people		
**Acceptability**[26,34,35,38]	The extent to which the intervention is judged as suitable, satisfying or attractive by different stakeholder groups (e.g. patients, providers or politicians). The acceptability depends on people their norms, beliefs and values [26,40].	**0** the intervention is not accepted by some people and it is not likely that this can be changed	8.67 *(5–15)*	10
		**1** the intervention is not accepted by some people but it is likely that this can be changed with some extra effort (e.g. special education)		
		**2** the intervention is accepted by almost all people		
**Cost-effectiveness**[31,32,35,37,40]	The capacity to produce the maximum output for a given monetary input [25].	**0** not cost-effective (e.g. costs per gained healthy life year are above 3*Gross Domestic Product (GDP) per capita)	12.4 *(0–25)*	15
		**1** cost-effective (e.g. costs per gained healthy life year are below 3*GDP per capita)		
		**2** highly cost-effective (e.g. costs per gained healthy life year are below 1*GDP per capita) [41]		
**Technical complexity**[26,34]	Other types of inputs required in addition to monetary nputs to implement and to keep providing the intervention. (These include human resource requirements, both quantitative and qualitative, and organizational requirements. The potential to integrate the intervention into an already existing health system should also be taken into account [26].	Ability to train and deliver all clinical and organizational requirements to run the intervention.	8.67 *(5–15)*	10
		**0** poor ability		
		**1** moderately good ability		
		**2** good ability		
**Affordability**[26,31,34,35,38]	The monetary input (e.g. capital investments and operational costs) required from the health system to implement and to keep providing the intervention [26].	**0** poor affordability (e.g. costs > 1 US$ per capita)	8.47 *(0–20)*	10
		**1** moderate affordability (e.g. costs > 0.50 ≤ 1 US$ per capita)		
		**2** good affordability (e.g. costs ≤ 0.50 US$ per capita) [26]		
**Safety**[31,34]	Safety is the practical certainty that adverse effects to patients or providers will not result from exposure to an intervention under defined circumstances [27].	**0** there is a risk of severe adverse effects (life threatening) to patients or a risk of adverse effects (of any kind) to providers	7.87 *(0–15)*	10
		**1** there is a risk of mild adverse effects to patients		
		**2** there is no risk or a risk of very mild adverse effects (adverse effects which will completely recover within a month) to patients		
**Geographical coverage**[26,32,34,35]	The ability of the intervention to be reached by the target population, independent of their living place [26].	**0** the intervention does not cover (most) people who live far away from cities.	5.47 *(0 – 13)*	5
		**1** the intervention does not cover some people who live far away from cities.		
		**2** the intervention covers (almost) all people		
**Accessibility**[32,37]	Patients with a different socioeconomic status or a different income should be able to make equal use of the intervention [32].	**0** the intervention is not accessible to many patients	10.6 *(0 – 20)*	13
		**1** the intervention is not accessible to some patients		
		**2** the intervention is accessible to (almost) all patients		

*weights were calculated by asking participants to divide 100 points over the criteria according to their relative importance for the evaluation of breast cancer interventions.

NOTE: References were used to identify the criteria in first instance. The Delphi study may have resulted in adaptations in definitions or scoring scales than originally found in the literature.

### Third round

All participants agreed that the list after the second round covered the most relevant criteria for the prioritization of breast cancer interventions. Three participants mentioned, however, that some criteria might be still overlapping. As one participant noted: “Doing the relative weighting exercise above, I realized that some criteria are overlapping and it was difficult to assess independent relative weights to them; for example, ‘effectiveness’ and ‘quality of the evidence’ are inseparable whereas we would not perhaps say something is effective if the quality of the evidence is weak”. There were also doubts about ‘cost-effectiveness’ being covered by the ‘affordability’ and ‘effectiveness’ and about ‘safety’ being covered by ‘effectiveness’ and ‘geographical coverage’ being covered by ‘effectiveness’. The criterion ‘geographical coverage’ was rated relatively low in its importance for the evaluation of breast cancer interventions, followed by ‘safety’ and ‘affordability’, respectively. The importance of the criterion ‘Effectiveness’ was rated highest (Table 2).

## Discussion

This study describes the development of a rating tool to measure the impact of breast cancer interventions based on multiple criteria in LMICs. Ten criteria, including definitions and potential scoring scales, have been indicated. The results of this study show that effectiveness, quality of the evidence, magnitude of individual health impact, acceptability, cost-effectiveness, technical complexity, affordability, safety, geographical coverage, and accessibility seem to be important principles in the selection of breast cancer control strategies. Although selecting and defining interventions and criteria for breast cancer control is context specific, we think that this rating tool can be a starting point for local policy makers as part of a broader, MCDA based, priority setting process.

### Use of the tool in a LMIC

**Figure 2:**
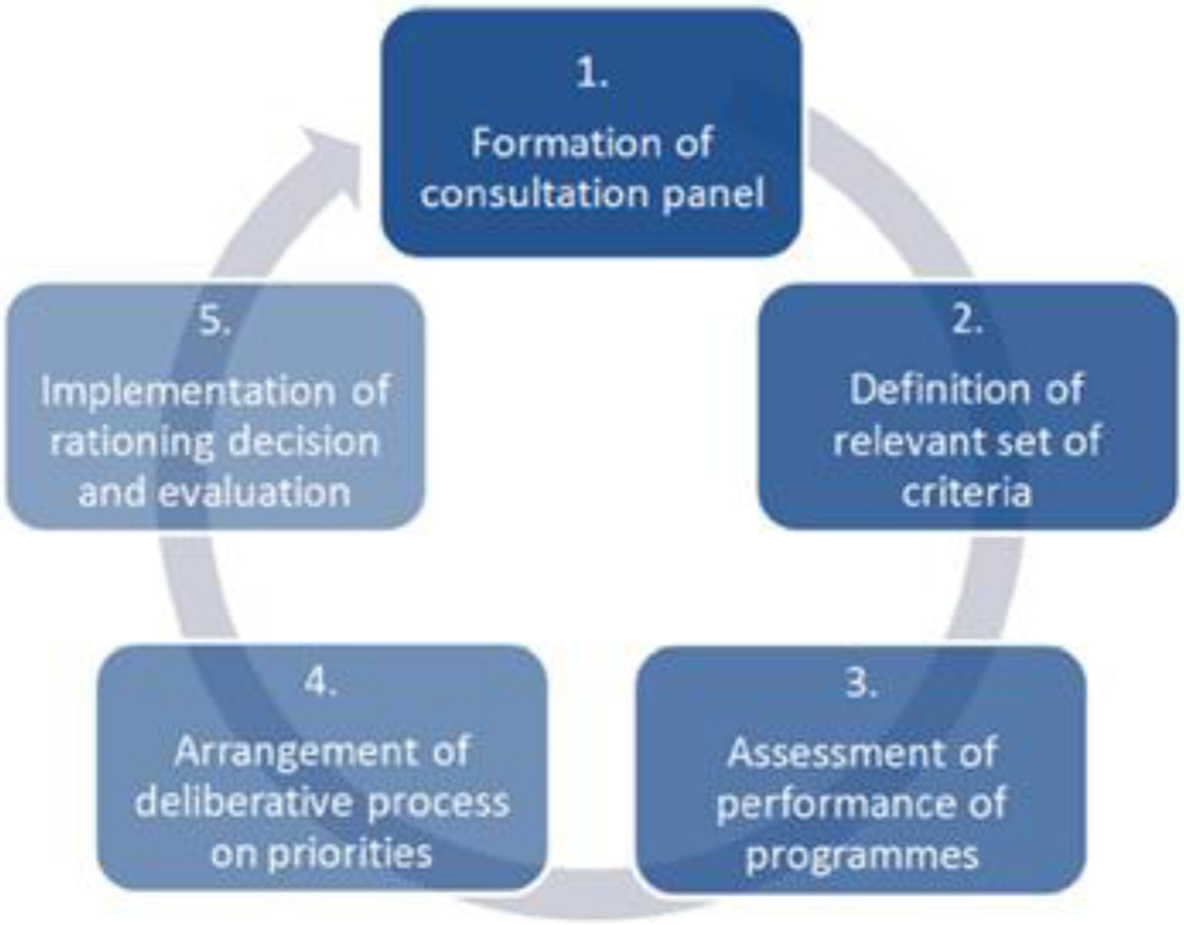
**Elements of a priority setting process based on MCDA [**[16]**].**

The tool could be used as part of the integrated MCDA and accountability for reasonableness (A4R) approach for priority setting, recently proposed by Baltussen et al. [16] (Figure 2). This new approach combines strong components of both frameworks and requires a set-up of a multi-stakeholder consultation panel (step one). In this way a democratic learning process is started in which stakeholders are involved in all steps of the priority setting. Compared to the stand-alone MCDA framework, this combined approach may increase the acceptance of decisions among stakeholders and the likelihood of implementation of prioritized programs. The rating tool can be part of step two and three (Figure 1) of the approach that aim to identify criteria for priority setting and assess (i.e. rate) the performance of interventions on the selected criteria.

An important next step in the local use of the rating tool is to investigate how the tool and its components are understood in LMICs in a pilot study. Users of the tool could for example select relevant stakeholders (e.g. patients, lay-people policy makers, caregivers, public health specialists) and establish a consultation panel (step 1). These stakeholders could discuss the interventions, criteria, the attitude of decision-makers against the criteria and scoring scales using democratic elements (e.g. Nominal Group Technique) (step 2). After the collection of all relevant (local) evidence, the users could use our tool as an input for a performance matrix (step 3), and then interpret and deliberate on the results of this matrix (step 4 and 5). Users should be well informed and plan enough time for this process, and should try to ensure that the tool is perceived as a simple and legitimate way to frame policy discussions in a more rapid and balanced manner. The potential of this tool could also be investigated for other cancers.

### Limitations of the study

Our study has a number of limitations. First, prior to the Delphi study, the expert panel made a selection of 9 criteria out of 33 criteria. This selection was based on overlap between criteria and whether criteria would be appropriate for the selection of breast cancer interventions. However, there is no certainty that personal preferences did not lead to bias in this selection.

Second, we used Delphi studies to define a list with core criteria including definitions and scoring scales. The Delphi method ensures participant anonymity and provides enough time to properly consider one’s own answers and those of others. However, the Delphi questionnaire may not allow for adequate elaboration on difficult concepts such as equity and social welfare. Also, Delphi questionnaires can be relatively time consuming, which may have partly caused 14 participants to withdraw from this study. We do not expect that these withdrawals biased the results because they varied in gender and type of expert and the number of comments remained high in each questionnaire.

Third, the wide variety of comments and views of the participants made us aware of the difficulties in developing a clear, consensus-based, non-overlapping criteria list and scoring scales. There are many possible compositions and definitions of criteria [31-33]. Besides there are many ways to divide a scoring scale into different categories and this also depends on the variability of the interventions that are considered (i.e. discriminative power of the scoring scale). Further research should focus on more informed contextualized categories for scoring scales.

The difficulty of avoiding overlap between criteria may be explained by a lack of a broader theory on the relationship between criteria. Some disagreement between participants remained until the end of the process, and some overlap was still suspected in the final criteria list. These potential overlaps will need attention in the further development of this tool because criteria should preferentially be independent from each other [15,42]. Especially effectiveness has a risk of overlap with other criteria, like cost-effectiveness, safety and geographical coverage. Further overlap between criteria should be identified and distinctions should be made and clearly described in the definitions.

### Limitations of the tool

The tool also has some practical limitations that one should be aware of. First, the tool does not provide guidance to convert the performance matrix into a final prioritization of interventions. This tool stops at rating interventions after which a choice should be made based on a democratic learning process (Figure 1). This tool does not facilitate a democratic learning process, which makes it less likely that good rated interventions are implemented. The accountability for reasonableness framework (A4R) is successful in introducing such a learning process [43]. We recommend making the tool part of the integrated MCDA A4R approach for priority setting in health as proposed by Baltussen et al. [16], however local capacity should be present or established to facilitate such a complete process.

Second, the proposed rating tool is based on decision-maker values and preferences while the views of other stakeholder groups are also considered important in priority setting exercises. Different stakeholder groups are likely to have different preferences for criteria [22,44]. This limitation of the tool could be solved while applying the tool in a local setting. At that stage, other stakeholder groups (like patients, the public, and health care workers) can be asked to comment on the relevance of the criteria included in the tool and the relative importance and the tool can be adapted accordingly.

Third, there are limitations to the collection of information, and it may sometimes be difficult to assess interventions on certain criteria. This is however a problem across the field of health priority setting and we recommend to be transparent on the available evidence and its quality. A sensitivity analysis may help to give insight in the uncertainty of the scoring performances of intervention options. In this way, quality of evidence is not used as a single criterion but as an uncertainty factor per criterion per intervention [45].

## Conclusion

This study describes the development of a rating tool to assess the impact of breast cancer interventions on multiple criteria. This tool may be a starting point for local decision makers that would like to conduct multi criteria decision analysis to set priorities for breast cancer control.

## Competing interests

The authors declare that they have no competing interests. The views expressed in this paper are those of the authors, and the funding organization has had no influence on them.

## Authors’ contributions

KV and SZ made substantial contributions to the conception and design of the study, acquisition of data, and analysis and interpretation of data. They also participated in the expert panel and drafted the manuscript. JL contributed to the design and methodology of the study and participated in the expert panel. NT assisted in the design of the study and has been involved in revising the manuscript critically for important intellectual content. All authors have read and approved the final manuscript.

## Supplementary Material

Additional file 1 **Key considerations for the development of the rating tool.** In this additional file we elucidate on the key considerations made for development of the rating tool. Click here for file
