# Supplementary material for: Multi-criteria decision analysis of breast cancer control in low- and middle- income countries: development of a rating tool for policy makers
Source: Cost Eff Resour Alloc. 2014 May 17;12:13. doi: 10.1186/1478-7547-12-13 (PMC4031156; doi:10.1186/1478-7547-12-13)
Supplement: Additional file 1 — Key considerations for the development of the rating tool. In this additional file we elucidate on the key considerations made for development of the rating tool. [file 1478-7547-12-13-S1.pdf]

Additional file 1

Key considerations for determining the step-by-step approach of the rating tool were obtained from '*The workbook on how to develop a priority setting framework*' provided by the South Australian Department of Health [1]. This workbook is developed for and used by the Clinical Senate of South Australia, a clinical advisory group to the Minister and Department of Health, to fairly assign resources to all kinds of medical interventions. The expert panel discussed key considerations for the development of the tool, taking the specific decision-making context into consideration. The key considerations can be found in Table 1. The discussion of the expert panel on each key consideration is elucidated further below.

Table 1. Key considerations for the development of the breast cancer rating tool

	**Consideration**	**Response of expert panel**<br>*(for this tool only)*	**Basis of consideration**
**1**	Whose values should inform the criteria?	Local policy-makers who are directly involved in the decision making process. The general public, patients and caregivers may be included in future analyses.	Setting in which the tool will be used and representativeness of the decision-makers.
**2**	How many criteria?	Probably between five and ten, depending on preferences of local policy-makers and local research capacity	Representativeness of decision-makers, feasibility of assessing the criteria and discriminative power of criteria.
**3**	What types of criteria?	All types of criteria that relate to the WHO health systems framework	Scope of the WHO
**4**	How should the criteria be established?	The tool will provide a predefined list based on literature reviews, followed by a Delphi study. Local decision-makers should discuss this list and add or	Consistency and ease of use of the tool.

		remove criteria if preferred.	
**5**	What process should be used in evaluating the options?	Assessment of options (intervention packages) should be presented in a performance matrix showing options in rows and their performance on each criterion in a column. Options will be evaluated on scoring scales with three categories. These scoring scales will be added to the predefined criteria list and also developed by a literature study, followed by a Delphi study. Local decision-makers may adapt the scoring scales to local circumstances. The options themselves should be clearly defined for each local situation and will consist of packages of interventions.	Ease of use and discriminative power
**6**	Who should be involved in evaluating the options?	At least two researchers should evaluate the options. Decision makers, the general public, patients and caregivers should be included in the local analyses.	Quality of the analysis.
**7**	Should the criteria be weighted?	Yes, decision-makers can assign their own weights according to their objectives and value trade-offs.	Transparency of decision-making process and distinctiveness of criteria

Adopted from the Clinical Senate of South Australia, advisory group to the Minister and Department of Health

1. Whose values should inform the criteria?

Many studies describe MCDA as modeling the objectives and value trade-offs of decision-makers to make the prioritization of alternatives more comparative and transparent [2, 3]. From this perspective, criteria will be constituted by the decision-makers. However, other studies call for inclusion of other stakeholders as well [4, 5]. Ideally, policy makers should be able to represent these stakeholders; however, in practice they may have other, stronger interests. Because the tool is developed from a

WHO perspective, it aims to be used in collaboration with member states on a national level. Therefore, the expert panel decided that in the first instance, the criteria for this specific tool should be informed by local policy-makers who are directly involved with the specific decision. It may however be locally decided that other stakeholders will be involved as well. To support this decision, future analyses may investigate differences in values and decisions of other stakeholders such as the general public, patients, and caregivers.

2. How many criteria?

How many criteria should be used in MCDA is debatable. Too many criteria would result in diluting the impact of individual criteria. Too few criteria would result in overlook important aspects for decision-making. This choice should depend on the discriminative power of criteria (i.e. depends on the interventions to be assessed). Various studies have included between three and fourteen criteria [6-8]. Taking the ease of use and feasibility into account, the expert panel concluded that for this tool, a short list of criteria would be more appropriate than a long and detailed list (probably five to ten criteria in LMICs). The final decision regarding the number and type of criteria should always involve preferences of stakeholders. Moreover, obtaining and analyzing sufficient evidence for each combination of criterion and intervention should be feasible. Hence, the number of relevant criteria in the local setting should furthermore depend on the capacity to assess the criterion and whether a criterion has discriminative power. This should take into consideration existing criteria used in the field to which the tool will be applied.

3. What types of criteria?

Most recognized types of criteria are medical, political, ethical, and economic [3]. MCDA generally includes all types of criteria related to principles determining priority-setting policies or standards [3]. This study stays in line with that concept as long as criteria are related to the scope of the WHO, i.e., the WHO health systems framework [9,10]. Therefore, we considered all criteria associated with the health system goals (improved health (level and equity), improved efficiency, responsiveness, social and financial risk protection), and all criteria that relate to the ‘inputs’ of health systems (service delivery, health workforce, information, medical products, vaccines and technologies, health financing, and leadership/governance).

4. How should the criteria be established?

In MCDA, decision-makers typically inform criteria, for example through focus group discussions [3, 11]. However, according to Baltussen et al., this method carries a risk of omitting relevant criteria since it is hard for decision-makers to think of all health goals and distribution issues as are mentioned in the literature [11]. Therefore, they suggested an alternative method in which a list of relevant criteria is predefined, based on present experience and the literature. Such a list reduces the risk of criteria omission and improves the consistency of criteria being considered. Moreover, predefined definitions and descriptions of criteria are expected to improve the understanding of the underlying concepts of each criterion. Because a predefined list also saves time and effort during analyses, the expert panel decided that the tool should be based on such a predefined list, with definitions and descriptions of criteria relevant for prioritizing breast cancer interventions. This list was developed by the Delphi study described in the article.

Subsequently, during the analysis, the list needs to be discussed and evaluated with local decision-makers to ensure that it is appropriate for the specific context and interventions (e.g., through focus group discussions, Delphi studies, or nominal group techniques). Based on these discussions, local decision-makers may decide to add or remove criteria from the list or to adjust criteria definitions.

5. What process should be used in evaluating the options?

Defining interventions

Defining interventions requires preparations similar to defining the criteria. When, for example, an intervention is not specified with regard to a certain target population or area, it would be impossible to score this intervention on the criterion ‘equity’ or ‘geographical coverage’. Because the rationale of the tool is to facilitate prioritization of interventions along the continuum of breast cancer care, different types of preventive, early detection, diagnostic, treatment, and palliative options may be assessed. These interventions can be designed for specific high-risk patients or selected communities or can have very generic features for the entire population. The type of interventions (specific vs. generic, or preventive vs. palliative) can influence their responsiveness to the different criteria; hence, the expert panel decided that very specific interventions (i.e. a single drug agent) should be combined in a more

comprehensive intervention package comprising of prevention, early detection, diagnoses, treatment and palliation elements. These packages should also prevent unrealistic or unethical situations in which, for example, only screening interventions are prioritized while no treatment is available. If intervention features show a high variation, comprehensive intervention packages should first be defined.

Assessing interventions

Baltussen and Niessen described how the scoring performances can be presented by a performance matrix, which is a standard feature of every multi-criteria analysis [2, 12, 13]. When using the tool, a performance matrix should be composed in which each row presents an intervention package and each column presents a criterion. The expected consequences of each intervention package on each criterion should then be assigned a score which is higher in case the intervention packages is more preferred on that criterion. To provide a directive for assigning scores, the expert panel decided to provide scoring scales with the criteria list.

Scoring scales

Scoring scales which are able to be used for priority setting criteria in health care are not strongly developed yet. Mullen et al. describe how scores can be obtained by simply assigning points, for example from zero to five [6]. Another method proposes developing simple ordinal categories for the criteria, to which scores can be assigned [12]. The experts decided that the latter method would be most appropriate for the tool because it offers an understandable and easy way of scoring options and clear definitions of categories will result in more objective evaluations.

To ensure comparability of the scoring performances on the criteria, scoring scales with similar structures are needed. Because this tool is a first concept which aims to be easy to use, the expert panel determined that scoring scales should be kept simple, with only three categories. For these categories, an intervention could score zero, one, or two points, and each category covers about one third of the total range of potential outcomes (normalized scale). The expert panel furthermore decided that predefining these scoring scales would allow for more consistent use of the tool. When applying the tool in LMICs, new scoring scales should be developed when criteria are added. Moreover,

because the cut-off points of scoring scales may differ by context, the definition of scoring scales should be discussed and modified with local experts when necessary. The development of scoring scales was done by the Delphi study as described in the article.

6. Who should be involved in evaluating the options?

According to Peacock et al., performance of the interventions should be determined by an advisory panel based on available evidence or technical judgments as necessary [3]. This advisory panel is not further explained in the literature but should probably include experts within the same decision-making context. The expert panel decided that for this tool, local researchers or experts should be evaluating interventions since the tool aims to restrict the need for specialized help. Local capacity and empowerment will however always be required [14]. So if necessary, local experts should be trained in critically assessing and using cutting-edge techniques. For this tool, the evaluation should be performed by at least two researchers to complement each other in technical expertise and to check and discuss one another’s findings. The evaluation should be based on local quantitative evidence as well as on local qualitative evidence revealed from interviews with local decision-makers, health care professionals, members of the general population, or other experts. International scientific evidence (e.g., on costs, cost-effectiveness, or effectiveness) might be used if local evidence is not available.

7. Should the criteria be weighted?

The performance matrix supports the decision maker in rating the options. Doing this in a qualitative manner may lead to the rating of intervention packages based on unjustified and non-transparent assumptions [2]. The expert panel therefore recommends quantitative analysis of the performance matrix. That means criteria should be assigned weights making it able to transparently correct for their relative importance [3].

A search through the literature shows up several methods for weighting criteria, like the swing weights method and discrete choice experiments [3, 11, 13]. Baltussen and Niessen describe a relatively simple method with which weights can be obtained by comparing the criteria to the most important criterion and subsequently calculating weights that sum up to 100 in total.

The expert panel decided that the last method would fit this first concept of the tool because this method is easy to use and understand. However, weighting criteria still allows for subjectivity arising

from differences in interpretation, understanding, and values of the participants. Therefore, the expert panel recommends that the provided weights and the provided scores should be assessed in sensitivity analyses (e.g. by including a range for weights and scores).

.

References

1. Leggat S: *Developing a Clinical Priority Setting Framework.* Melbourne: South Australian Department of Health, Adelaide & La Trobe University, School of Public Health; 2004.

2. Baltussen R, Niessen L: **Priority setting of health interventions: the need for multi-criteria decision analysis.** *Cost Eff Resour Alloc* 2006, **4**:14.

3. Peacock S, Mitton C, Bate A, McCoy B, Donaldson C: **Overcoming barriers to priority setting using interdisciplinary methods.** *Health Policy* 2009, **92**(Suppl 2-3):124-132.

4. Youngkong S, Baltussen R, Tantivess S, Koolman X, Teerawattananon Y: **Criteria for priority setting of HIV/AIDS interventions in Thailand: a discrete choice experiment.** *BMC Health Serv Res* 2010, **10**:197.

5. Youngkong S, Kapiriri L, Baltussen R: **Setting priorities for health interventions in developing countries: a review of empirical studies.** *Trop Med Int Health* 2009: **14**(Suppl 8):930-939.

6. Mullen PM: **Quantifying priorities in healthcare: transparency or illusion?** *Health Serv Manage Res* 2004, **17**(Suppl 1):47-58.

7. Golan O, Hansen P, Kaplan G, Tal O: **Health technology prioritization: which criteria for prioritizing new technologies and what are their relative weights?** *Health Policy* 2011, **102**(Suppl 2-3):126-135.

8. Guindo LA, Wagner M, Baltussen R, Rindress D, van Til J, Kind P, Goetghebeur MM: **From efficacy to equity: Literature review of decision criteria for resource allocation and healthcare decisionmaking.** *Cost Eff Resour Alloc* 2012, **10**(Suppl 1):9.

9. World Health Organization: *Everybody's business: Strenthening health systems to improve health outcomes: WHO's framework for action.* Geneva; 2007.

10. Tromp N, Baltussen R: **Mapping of multiple criteria for priority setting of health interventions: an aid for decision makers.** *BMC Health Serv Res* 2012, **12**:454.

11. Baltussen R, Youngkong S, Paolucci F, Niessen L: **Multi-criteria decision analysis to prioritize health interventions: Capitalizing on first experiences.** *Health Policy* 2010, **96**(Suppl 3):262-264.

12. Devlin NJ, Sussex J: *Incorporating multiple criteria in HTA. Methods and processes.* London: Office of Health Economics; 2011.

13. Dodgson JS, Spackman M, Pearman A, Phillips LD: **Multi-criteria analysis: a manual.** *Department for Communities and Local Government.* London; 2009.

14. Youngkong S, Baltussen R, Tantivess S, Mohara A, Teerawattananon Y: **Multicriteria decision analysis for including health interventions in the universal health coverage benefit package in Thailand.** *Value Health* 2012, **15**(Suppl 6):961-970.
